# O8.5. SCHIZOPHRENIA AND BIPOLAR DISORDER DIAGNOSIS PATTERNS: REAL-WORLD EVIDENCE FROM US CLAIMS DATABASES

**DOI:** 10.1093/schbul/sby015.241

**Published:** 2018-04-01

**Authors:** Mallik Greene, Tingjian Yan, Eunice Chang, Ann Hartry, Maelys Touya, Maxine Chen, Jennifer Munday

**Affiliations:** 1 Otsuka Pharmaceutical Development & Commercialization, Inc; 2 Partnership for Health Analytic Research, LLC; 3 Lundbeck; 4 Phar LLC

## Abstract

**Background:**

Schizophrenia and bipolar disorder (BD) are typically understood as separate and non-concurrent psychiatric disorders both in the clinical setting and in the DSM-V and ICD-10 classification systems. However, patients may experience both mood and schizophrenia symptoms simultaneously. Several studies have shown overlap between schizophrenia and BD symptoms, which may lead to diagnostic confusion. Additionally, molecular studies have confirmed that schizophrenia and BD share susceptibility genes. This study explored diagnosis patterns of patients with schizophrenia and/or type I bipolar disorder (BD-I) diagnoses in a real-world setting.

**Methods:**

This was a retrospective cohort study using Truven MarketScan® Commercial, Medicaid, and Medicare Supplemental databases from the study period 01/01/2012 and 06/30/2016. Patients were considered to have a diagnosis of schizophrenia if 1 inpatient claim or 2 outpatient claims for schizophrenia were identified within a selected identification period (01/01/2013 and 06/30/2015). BD-I was defined in an analogous way, and the following five mutually exclusive cohorts were defined: 1) schizophrenia (SCZ) alone (cohort I): newly diagnosed with schizophrenia alone (e.g., met the claims-based diagnostic criteria for schizophrenia, but not for BD-I), 2) BD-SCZ (cohort II): met BD-I criteria only in the year prior to meeting the schizophrenia criteria, 3) SCZ-BD (cohort III): met schizophrenia criteria only in the year prior to, or on the same day as, meeting BD-I criteria, 4) BD-SCZ-BD (cohort IV) met BD-I criteria both in the year before and the year after meeting the schizophrenia criteria, and 5) BD alone (cohort V): newly diagnosed with BD-I alone (e.g., met the claims-based diagnostic criteria for BD-I, but not for schizophrenia). Descriptive statistics are reported for all cohorts.

**Results:**

Of the 63,725 patients in the final analytic sample, 11.5% (n=7,336) had schizophrenia alone (cohort I), 7.7% (n=4,909) had a dual diagnosis (cohorts II-IV), and 80.8% (n=51,480) had BD-I alone (cohort V). The dual diagnosis patients included 1.0% (n=615) with BD-SCZ (cohort II), 2.8% (n=1,794) with SCZ-BD (cohort III), and 3.9% (n=2,500) with BD-SCZ-BD (cohort IV). Patients with different diagnosis patterns significantly differed in age, gender, and insurance type (p<.001). Considering the dual diagnosis cohorts, 927 received both diagnoses on the same day. Of those occurring on the same day, the majority (n=753) were on claims from the hospital/emergency department setting.

**Discussion:**

This analysis of real-world data found a sizable number of patients with dual diagnoses of schizophrenia and BD-I. Among all patients with either BD-I, schizophrenia, or both, about 2/3 as many met the criteria for both disorders as for schizophrenia alone. Fifteen percent of patients who met criteria for both did so on the same day, likely reflecting patients presenting to acute care exhibiting mixed features. A review of medical records would be useful to determine if dual diagnosis is more common than suspected, and claims data should be examined to determine if these patients differ sufficiently from those with a single diagnosis to warrant exclusion from single-disease cohorts.

